# Complement inhibitor Crry expression in mouse placenta is essential for maintaining normal blood pressure and fetal growth

**DOI:** 10.1371/journal.pone.0236968

**Published:** 2020-08-03

**Authors:** Manu Banadakoppa, Kathleen Pennington, Meena Balakrishnan, Chandra Yallampalli

**Affiliations:** Department of Obstetrics & Gynecology, Baylor College of Medicine, Houston, Texas, United States of America; Loma Linda University School of Medicine, UNITED STATES

## Abstract

Many circumstantial evidences from human and animal studies suggest that complement cascade dysregulation may play an important role in pregnancy associated complications including preeclampsia. Deletion of rodent specific complement inhibitor gene, Complement Receptor 1-related Gene/Protein y (Crry) produces embryonic lethal phenotype due to complement activation. It is not clear if decreased expression of Crry during pregnancy produces hypertensive phenotype. We downregulated Crry in placenta by injecting inducible lentivialshRNA vectors into uterine horn of pregnant C57BL/6 mice at the time of blastocyst hatching. Placenta specific downregulation of Crry without significant loss of embryos was achieved upon induction of shRNA using an optimal doxycycline dose at mid gestation. Crry downregulation resulted in placental complement deposition. Late-gestation measurements showed that fetal weights were reduced and blood pressure increased in pregnant mice upon downregulation of Crry suggesting a critical role for Crry in fetal growth and blood pressure regulation.

## Introduction

Complement cascade consisting of more than 30 proteins is a part of innate immune system. Complement is typically activated through three distinct pathways, the “classical”, the “lectin” and the “alternative” pathways [1]. All three pathways converge at the level of C3 activation and proceed to form a common cellulolytic pore like structure, membrane attack complex (MAC) in cell membrane [2]. Both in humans and mice, self-cells are protected from complement attack by three membrane-bound proteins, decay accelerating factor (DAF or CD55), membrane cofactor protein (MCP or CD46) and MAC inhibitory protein (CD59) [3–5]. The early steps of complement are regulated by DAF and MCP whereas CD59 regulates the terminal steps. In addition to DAF, MCP and CD59, complement system of rodents contains a specific inhibitor, Complement Receptor 1-related Gene/Protein y (Crry). The expression of Crry has been observed in developing murine placenta [6].

Through complement activation or independently, components of the complement system such as C3, MBL and C1q play an important role in the success of normal pregnancy [7–9]. Further, normal pregnancy is a state of increased complement activation compared to non-pregnant women [10,11]. Increase in complement activation over the levels found in normal pregnancies are associated with pregnancy complications such as fetal growth restriction, spontaneous miscarriage, preterm birth and preeclampsia [12–14]. In mice, Crry gene deletion resulted in embryonic lethality. The Crry^-/-^ embryos died in utero by 9.5 days post coitus (dpc) due to complement activation [6]. The embryonic lethal phenotype was rescued when the Crry knock out was introduced with deficiency of C3 (C3^-/-^), or factor B (fB^-/-^) background [15], further confirming that Crry gene deletion caused fetal demise through complement activation.

Recently, the complement cascade has been implicated in the pathophysiology of preeclampsia. In preeclampsia patients, levels of complement activation by-products (C3a, Bb, C5a, and terminal complex MAC) are increased in circulation compared to normotensive pregnancies [10,16–19]. Increased complement activation (complement deposition) locally on placenta was also observed in preeclampsia patients [20, 21]. In addition, we have shown earlier that levels of membrane bound regulators on placentas in case of idiopathic miscarriage were decreased, by-products of complement activation in amniotic fluid in case of early-onset preeclampsia were elevated and complement activation on isolated human syncytial trophoblast cells induced dose dependent release of preeclampsia associated antiangiogenic molecule sFLT111, 12 [22]. Further, gene mutations in membrane bound regulators and circulating effector proteins have been reported to be associated with preeclampsia [23,24]. Complement has been implicated in several putative preeclampsia mouse models also. DBA/2 mated CBA/J pregnant mice have been used as a preeclampsia model since they show many features of human preeclampsia. However, DBA/2 x CBA/J pregnancies do not result in hypertension which is the main clinical feature of human preeclampsia. Nevertheless, site specific inhibition of complement activation prevented features of preeclampsia such as oxidative stress, placental dysfunction and renal pathology in DBA/2 x CBA/J pregnancies [25]. In the mildly hypertensive mouse model BPH/5 which recapitulates several preeclampsia features during pregnancy, site specific blocking of complement activation prevented preeclampsia symptoms [26]. In an autoantibody mediated mouse model of preeclampsia it has been shown that complement C3a receptor activation contributed to the pathogenesis of preeclampsia [27].

Thus, many circumstantial evidence implicate the complement cascade in the pathophysiology of preeclampsia. However, it is not clear if there is a direct relationship between complement activation and preeclampsia pathophysiology, mainly hypertensive phenotype. An attractive approach to this end would be to induce complement activation by abrogating the expression of membrane bound complement regulators on placenta after implantation. Among the key membrane bound complement regulators in mice, expression of MCP was observed only in testis [15,28]. The DAF was found to be expressed on placenta at around 10.5 dpc, much later than the expression of Crry [6,15]. DAF^-/-^ homozygous mice have been generated previously [29] suggesting that DAF knockout may not result in reproductive abnormalities. It is possible that the DAF function in placenta is redundant. Therefore, Crry is an attractive target to induce complement activation at the feto-maternal interface in mice. Since knockout of Crry is lethal, we hypothesized that depletion of Crry on placenta rather than complete abrogation would induce complement activation without lethal phenotype. In this study by using a shRNA to downregulate placental Crry without causing substantial embryo loss we show that Crry downregulation is associated with placental complement activation, reduced fetal growth and hypertension in pregnant mice.

## Materials and methods

### Crry shRNA-vector preparation

Doxycycline inducible “SMARTvector Lentiviral” vectors enclosing Crry shRNA and non-target shRNA were purchased from Horizon discovery (Lafayette, CO, USA). Lentiviral particles enclosing these vectors were produced in our laboratory using kit as per the instructions provided by the manufacturer (Horizon discovery). Approval from institutional biosafety committee at Baylor College of Medicine was obtained for these procedures. The functional titer of lentiviral particles were determined by transducing into HEK293 cells before injecting into mice. Adjustments to the volume were made to account for the variations in transducing units between the batches of viral particles.

### Intrauterine injection of lentiviralshRNA vectors

All animal procedures were approved by the Baylor College of Medicine institutional animal care and use committee and performed in accordance with NIH Guide for the Care and Use of Laboratory Animals. Eight week old C57BL/6J female mice were obtained from Jackson Laboratory (Bar Harbor, ME, USA). Mice were mated to C57Bl/6J proven breeder males for five days and the day of observed copulatory plug was identified as day post coitus (dpc) 0.5. On 3.5 dpc, a small incision was made on the right abdominal side of pregnant mice under isoflurane anesthesia to access right uterine horn. Crry shRNA or non-target shRNA lentiviral vectors (1250 transducing units/20 μl KSOM) were then injected into right uterine horn. Abdominal wall was closed using bio absorbable suture and wound clip was applied to close the skin. Meloxicam injection (ID) was given as analgesic. On 10.5 dpc doxycycline was added to drinking water to induce the shRNAs.

### Western blot and qPCR analysis

Tissue lysates containing 20μg protein were electrophoresed on 5–20% (w/v) polyacrylamide gradient gels (Thermo Fisher, Grand Island, NY, USA) and transferred on to Immune-Blot PVDF membranes (Bio-Rad, Hercules, CA, USA). Expression levels of Crry and C3 were probed using rabbit monoclonal antiCD46 antibody (Abcam, Cambridge MA, USA, cat# 108307). A secondary antibody goat anti-rabbit IgG-HRP (Southern Biotech, USA) and pierce ECL Western blotting substrate kit (Thermo Fisher) were used to develop the membranes. The Western bands were visualized using Odyssey IR imaging system (LI-COR Biosciences, Lincoln, NE, USA).

### Immunofluorescence analysis

Immunofluorescence was performed on OCT-embedded frozen sections after fixing with ice-cold Methanol for 10 minutes at -20°C. The sections then were incubated with 3% Goat serum in PBS-Tween (0.01%) for 1 hour at room temperature in a humidified chamber to block nonspecific antibody binding. For co-staining, the primary antibodies, mouse anti-Crry antibody (Hycult Biotech, Wayne, PA, USA) and rabbit polyclonal anti-C3 (Abcam, Cambridge, MA, USA) were applied at 1:200 dilution overnight, followed by goat anti-mouse TX-red and anti-rabbit IgG Alexa Fluor 488 (Molecular Probes, Eugene, OR, USA) for 1 hour at room temperature in a humidified chamber. Mouse and rabbit IgG (ready to use) (Dako, Glostrup, Denmark) was used as a negative control. Slides were then counterstained with Hoechst 33342 (Molecular Probes). Anti-fade mounting medium (Dako) was applied and slides were viewed and captured with a constant exposure time and aperture using a single threshold value under Olympus BX51 epifluorescence microscope, and images were recorded by a DP70 Digital Camera (Olympus Optical Co. Ltd., Tokyo, Japan). Subsequently, images were analyzed using ImageJ software and the numerical output of average intensity per nuclei was calculated.

### Blood pressure (BP) measurement and tissue collection

On 17.5 dpc, BP (systolic, diastolic and mean) was measured by tail cuff method in non-anesthetized mice using new 8-channel CODA system (Kent Scientific Corporation, Torrington, CT, USA). During BP measurement mice were restrained in the nose cone holders with unrestricted breathing and clear visibility. To minimize restrain anxiety the mice were conditioned for 20 minutes daily for 3 days before measuring BP. An occlusion tail cuff and volume pressure recording (VPR) senor were gently inserted onto the tails of mice placed on a pre-warmed (37°C) platform. Recordings were performed in three sessions with 15 cycles in each session. The readings were averaged and averages of 3 sessions were again averaged to get the final reading.

After BP measurement, mice were euthanized and blood was collected to obtain serum. Placental and fetal weights were recorded. Placenta, kidney, liver, and uterine tissue were collected for Western blot, qPCR and immunofluorescence analysis.

### Statistics

For all the experiments data was presented as mean ± SEM. Differences between two groups were analyzed by student t test followed by Mann-Whitney whereas ordinary ANOVA was used for more than two groups. A *p* value of less than 0.05 was considered to be significant. GraphPad prism (GraphPad Software, San Diego, CA, USA) was used for statistical analysis.

## Results

### Intrauterine injection of CrryshRNA down regulated placental Crry

**Fig 1:**
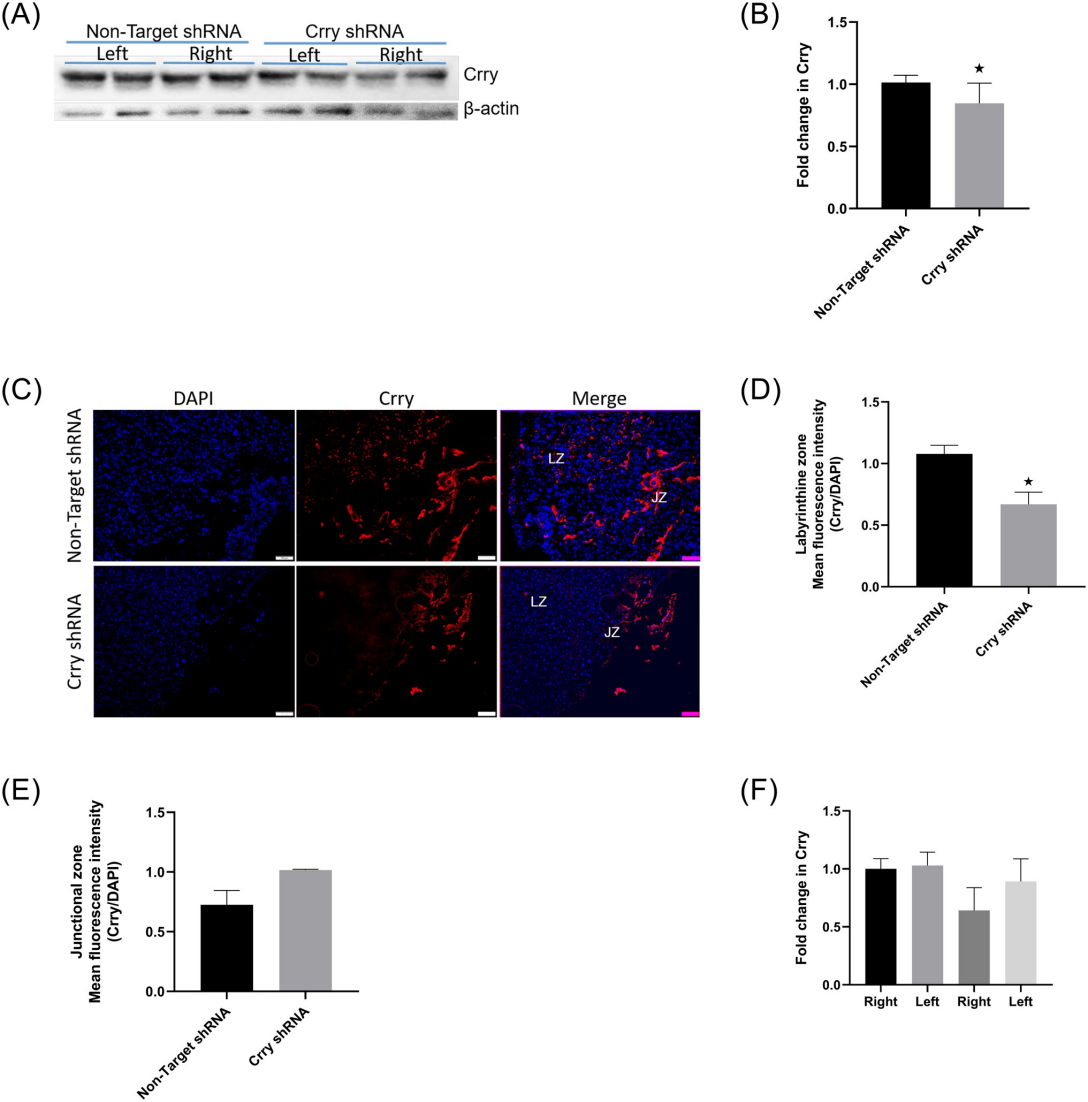
Downregulation of placental Crry. Lentiviral vectors enclosing Crry or non-target shRNA were injected into right uterine horn of pregnant mice (n = 5–7) on 3.5 dpc, shRNA was induced on 10.5 dpc and placental expression of Crry on 17.5 dpc was analyzed. A) Representative Western blot showing downregulation of Crry in CrryshRNA mice. In each group placentas from both left and right uterine horn were used in the blot. B) Density analysis of Crry Western bands showing about 30% reduction in Crry levels in CrryshRNA mice compared to control mice (*p<*0.021). C) Representative immunofluorescence images showing reduced Crry expression in CrryshRNA mice. D) Semi quantitation of fluorescence intensity revealed significant downregulation (*p* = 0.04) of Crry in the labyrinthine zone of CrryshRNA mice compared to control mice. E) There was no change in fluorescence intensity in junctional zone between the two groups. F) In CrryshRNA mice downregulation of Crry on placentas from right uterine horn showed trend of increase compared to those from left uterine horn.

We injected lentiviral particles enclosing either non-target (control group) or CrryshRNA vectors into right uterine horn of pregnant C57BL/6 mice (n = 5–7) around the time of blastocyst hatching (3.5 dpc). Since reduced expression of Crry before 9.5 dpc could affect embryo survival, we induced shRNA after 10.5 dpc. Initially, we titrated 3 doses of doxycycline (25, 75 and 500 μg/mL) to determine an optimum dose at which there was minimal embryo loss (S1 Fig). A doxycycline dose of 75 μg/mL resulted in downregulation of Crry with minimal embryo loss in CrryshRNA injected mice and therefore, this dose was used in the subsequent experiments. Although embryo survival rate was better at 25 μg/mL in CrryshRNA group, the Crry downregulation was not different from control mice. Since preeclampsia symptoms in humans appear in the third trimester we sought to examine the mice Crry and complement pathway in late gestation. Western blot analysis revealed that at 17.5 dpc, placental Crry expression levels were reduced by about 30% (Fig 1A and 1B) in CrryshRNA group compared to control group (*p<*0.021). In addition, immunofluorescence imaging (Fig 1C) further confirmed that Crry expression levels were reduced on placentas from Crry shRNA mice compared to control group. Immunofluorescence imaging further revealed that placental labyrinthine zone Crry expression levels were significantly different (*p* = 0.04) between Crry shRNA mice and control mice (Fig 1D). However, junctional zone Crry levels were not significantly different between the two groups (Fig 1E).

Western blot analysis further showed that in control mice there was no difference in the levels of Crry expression on placentas from left uterine horn compared to those from right uterine horn. In CrryshRNA mice, Crry expression levels showed a gradient. We observed a trend of increase in the magnitude of decrease in Crry levels on the placentas from right uterine horn compared to those from the left uterine horn in CrryshRNA mice (Fig 1F). However, the difference was not statistically significant.

**Fig 2:**
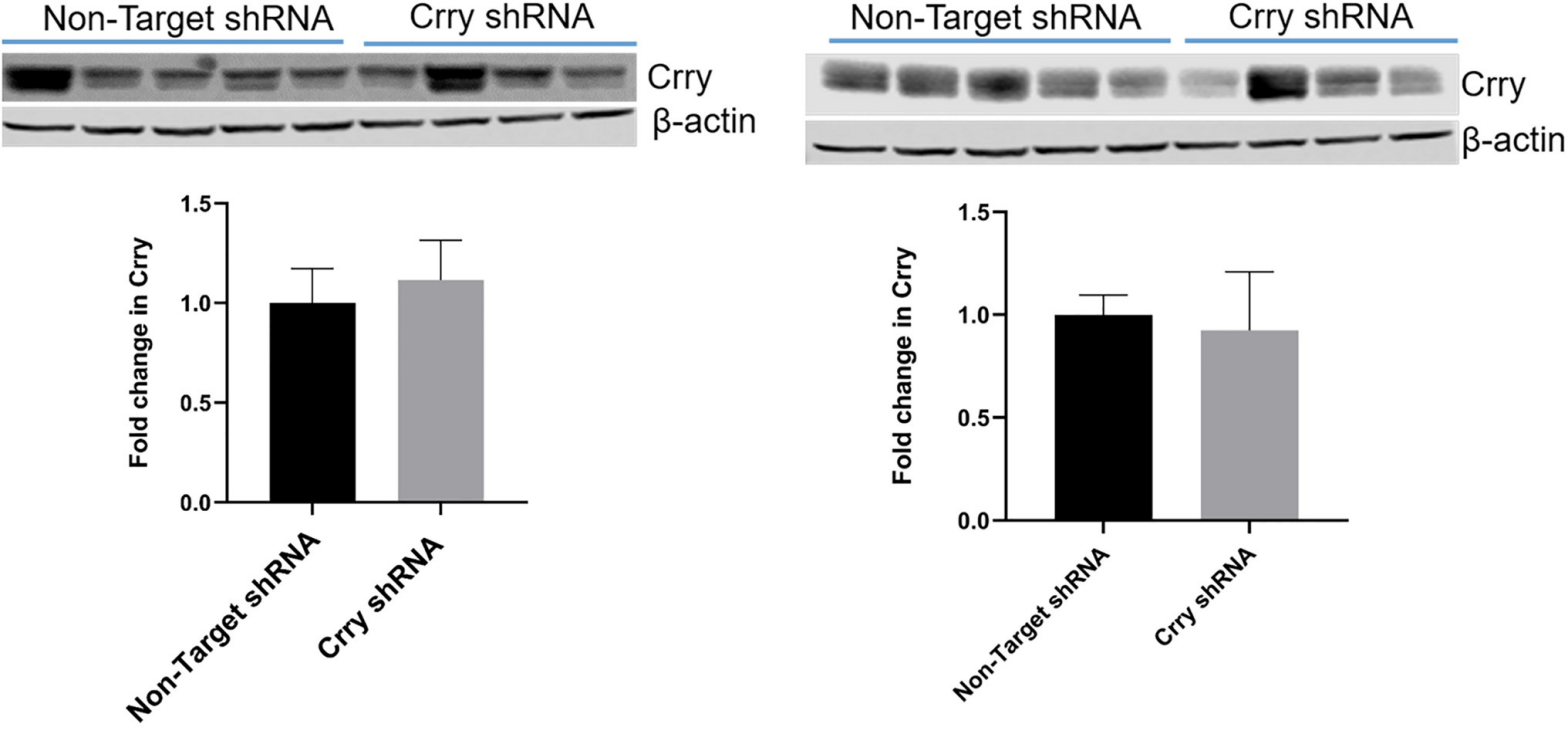
Decrease in Crry expression was placenta specific. Lentiviral vectors enclosing Crry or non-target shRNA were injected into right uterine horn of pregnant mice (n = 5–7) on 3.5 dpc, shRNA was induced on 10.5 dpc. Expression of Crry on 17.5 dpc in uterine tissue and liver was analyzed. Left panel is representative Western blot showing Crry levels in uterine tissue and density analysis. Right panel is representative Western blot showing Crry levels in liver and density analysis.

Western blot analysis of Crry expression levels in uterus (Fig 2, left panel) and liver (Fig 2, right panel) showed that there was no difference between the two groups suggesting that Crry expression decreased in a placenta specific manner.

Overall, these results suggested that injecting vectors enclosing shRNA directly into uterine horn at the time of blastocyst hatching followed by shRNA induction starting at 10.5 dpc downregulated Crry expression levels on placentas. The Crry downregulation showed a gradient with more decreases in uterine horn that received vectors than in the contralateral horn. The decrease in the Crry expression was specific to labyrinthine zone. Further, decrease in the Crry expression appeared to be placenta specific since levels of Crry in the uterus and the liver were not different between CrryshRNA injected and control mice.

### Down regulation of Crry resulted in placental complement deposition

**Fig 3:**
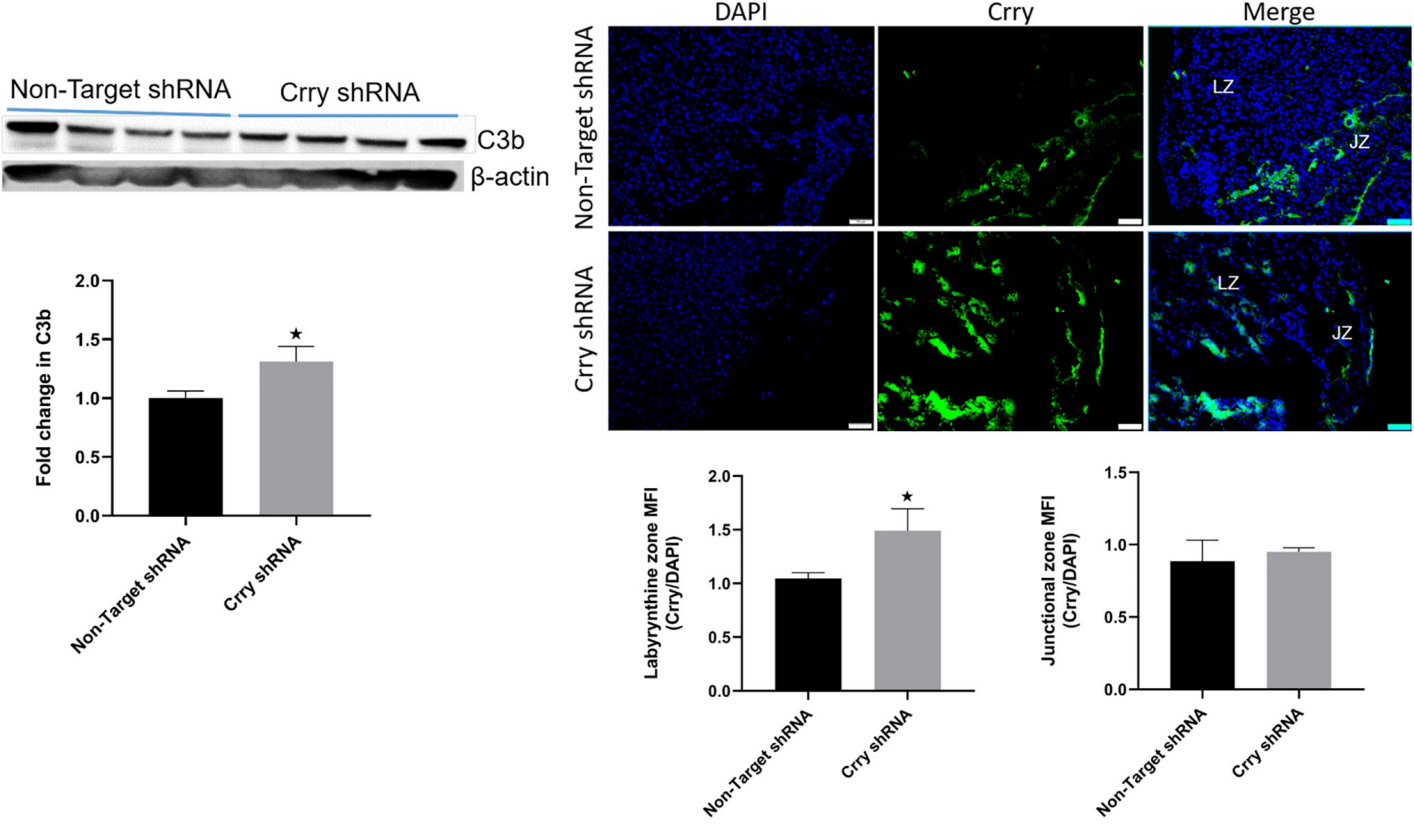
Crry downregulation triggered complement deposition on placenta. Placental complement deposition on 17.5 dpc was measured after shRNA induction on 10.5 dpc in pregnant mice (n = 5–7). Left panel is representative Western blot showing placental C3b deposition levels and density analysis of placental C3b Western blots showing significantly (*p* = 0.03) higher deposition in CrryshRNA mice compared to control group. Right panel is representative immunofluorescence images showing increased C3b deposition in CrryshRNA mice and semi-quantitation of fluorescence intensity showing significantly higher C3b deposition (*p* = 0.04) in the labyrinthine zone of CrryshRNA mice compared to control mice. There was no change in fluorescence intensity in junctional zone between the two groups.

Western blot analysis revealed that at 17.5 dpc, deposition of C3b was significantly higher (*p* = 0.03) on placentas from CrryshRNA compared to those from control mice. About 30% more C3b was deposited on placentas from CrryshRNA mice than those from control mice (Fig 3, left panel). Immunofluorescence staining further confirmed that higher levels of C3b was deposited on placentas from CrryshRNA mice compared to those from control mice (Fig 3, right panel). Consistent with Crry expression levels, the immunofluorescence imaging also showed that the C3b deposition was significantly higher (*p* = 0.04) in labyrinthine zone of placentas from CrryshRNA mice Deposition of C3b in junctional zone was not different between the two groups. Taken together, the data showed that complement activation on placentas increased in CrryshRNA mice due to the downregulation of Crry.

### C3 production up regulated in the liver to maintain serum levels

**Fig 4:**
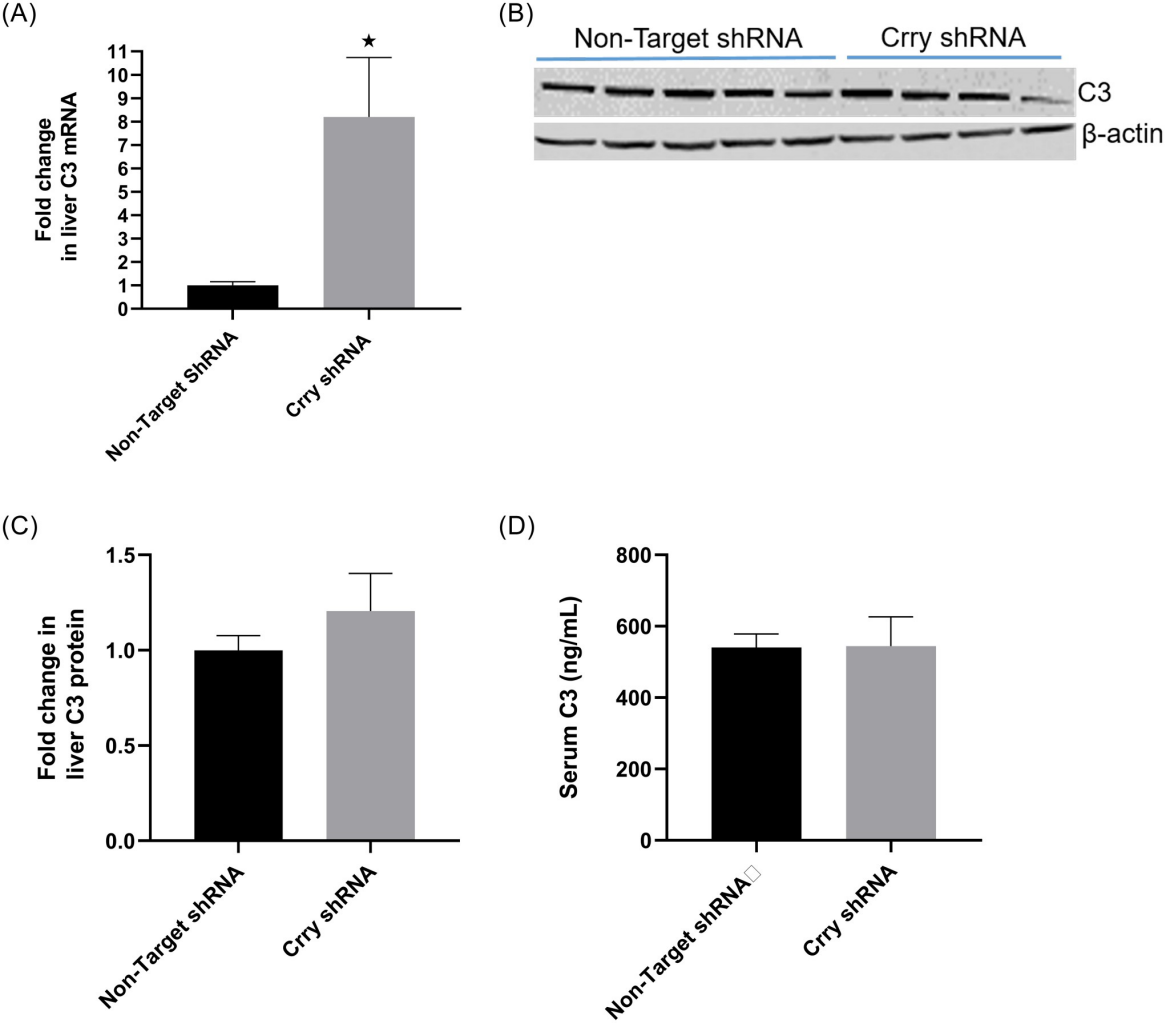
Feed-back upregulation of C3 in liver. Liver and serum C3 level on 17.5 dpc in pregnant mice (n = 5–7) were measured. A) RT-qPCR showing significantly (*p* = 0.012) elevated C3 transcript levels in liver from CrryshRNA mice compared to control mice. B) Representative Western blot showing C3 expression level in liver C) Density analysis of liver C3 Western bands reveled no difference in its levels between CrryshRNA and control groups D) Serum C3 levels measured by ELISA showing no difference between CrryshRNA and control mice.

Since liver is the major source for circulating C3, we speculated that increased consumption of circulating C3 due to complement activation at placenta would stimulate C3 production in liver to replenish circulating levels. Therefore, we measured serum C3 levels and liver C3 mRNA and protein levels. RT-qPCR revealed that C3 transcript levels were significantly higher (*p* = 0.016) in the liver from CrryshRNA mice compared to control mice (Fig 4A). Western blot analysis revealed that liver C3 levels were not different between the CrryshRNA and control mice (Fig 4B and 4C). The ELISA results showed that serum C3 levels were not different between the two groups (Fig 4D). Taken together, these results suggested that liver C3 transcript levels were increased upon placental complement activation in CrryshRNA mice, and C3 protein levels were not higher corresponding to mRNA levels in CrryshRNA group indicating secretion of excess C3 into circulation. However, serum C3 levels were not higher in CrryshRNA group suggesting that there was a feed forward loop where C3 consumption at placenta depleted serum C3 levels resulting in upregulation in C3 production in liver and therefore, serum levels were replenished.

### Fetal weights decreased and blood pressure increased in CrryshRNA mice

**Fig 5:**
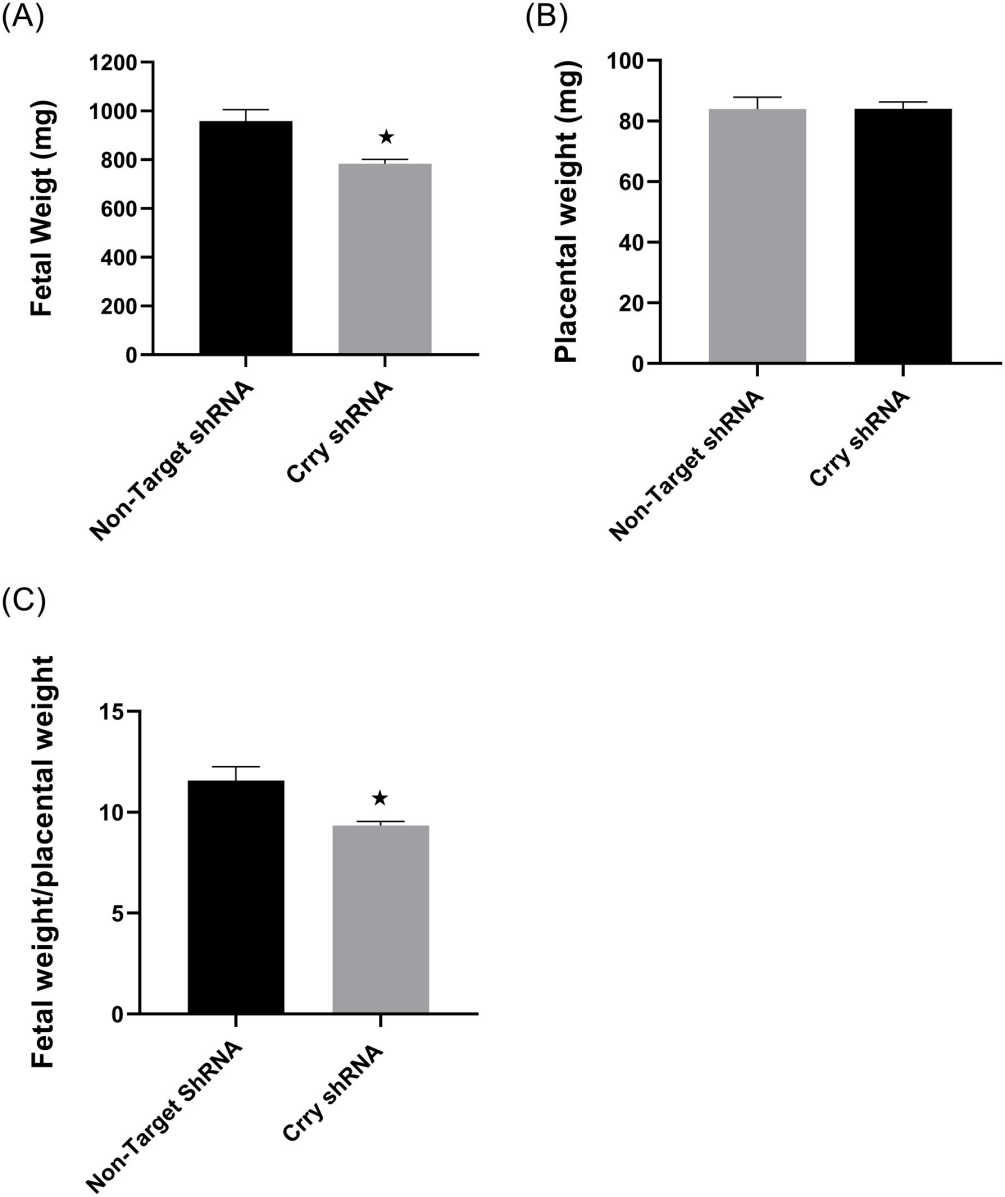
Decreased fetal weight in CrryshRNA mice. Fetal and placental weights were measured at 17.5 dpc soon after euthanasia (n = 5–7), A) Average fetal weight was significantly reduced (*p* = 0.048) in CrryshRNA mice compared to control group B) Average placental weight was not different between the two groups C) Fetal-placental weight ratio was significantly decreased (*p* = 0.045) in Crry shRNA mice compared to control mice.

The average litter size was not different between CrryshRNA and control mice (S2 Fig). The average fetal weight was significantly reduced (*p* = 0.048) in CrryshRNA mice (958.69±46.9 mg) compared to control group (783.69±18.02 mg) (Fig 5A). Placental weight was not significantly different (83.99±3.84 mg v/s 84.01±2.3 mg) between the two groups (Fig 5B). However, fetal-placental weight ratio was significantly decreased (*p* = 0.045) in Crry shRNA mice compared to control mice (Fig 5C) suggesting a reduced placental efficiency in CrryshRNA mice.

Further, the systolic, diastolic and mean blood pressures were significantly higher (*p* = 0.026, *p* = 0.008 and *p* = 0.008 respectively) in CrryshRNA mice compared to control mice (Table 1). Overall these data indicated that placental complement activation due to the downregulation of Crry decreased fetal growth and increased maternal blood pressure.

**Table 1:** Blood pressure elevated in CrryshRNA mice (n = 5–7).

Animal Group	Systolic mmHg	Diastolic mmHg	Mean mmHg
**Non-target shRNA**	133.95 ± 9.86	108.91 ± 7.2	118.08 ± 7.86
**Crry shRNA**	166.12 ± 4.08	135.07 ± 3.44	144.58 ± 3.36
***P***	0.026	0.008	0.008

## Discussion

We injected an inducible lentiviral vector enclosing shRNA targeted to Crry directly into right uterine horn in mice during the time of blastocyst hatching (3.5 dpc). The shRNA was induced starting at mid-gestation (10.5 dpc) using a dose of doxycycline that resulted in minimal loss of embryos. At late-gestation (17.5), we observed that Crry was downregulated in a placenta specific manner. With the optimal dose of doxycycline used Crry downregulation was about 30% compared to its expression levels in the control group. Further, the decrease in Crry was specific to labyrinthine zone. The downregulation of Crry resulted in increased complement activation on placentas from CrryshRNA mice. The C3 mRNA was upregulated in liver of CrryshRNA mice as a feed- forward response to decreased serum levels due to increased C3 turnover at fetal-maternal interface. Fetal weights were reduced and blood pressure increased in CrryshRNA mice suggesting a critical role for Crry in normal fetal growth and normal blood pressure. Since it is known that Crry plays an important role in the complement system homeostasis [30] and our data indicated an increased placental complement activation upon Crry downregulation, we suggest that the complement activation facilitated the effect of Crry downregulation on fetal growth and blood pressure.

Initially we attempted to employ published methods for placenta specific gene manipulation in rodents. Previously, several research groups used lentiviral vectors to manipulate genes in a placenta specific manner [31–33]. In these methods embryos were extracted from pregnant mice, zona pellucida were removed by chemical treatment and incubated with lentiviral vectors in vitro. Upon incubation, trophectoderm specific vector integration was observed [34]. These embryos were then transferred to timed pseudo pregnant female mice. We were unable to obtain pregnant mice with normal level of embryo survival using these methods. Since Crry depletion also gives embryonic lethal phenotype, we were unable to ascertain if the loss of embryos were due to Crry downregulation or methodology. Therefore, we decided to inject lentiviral vectors directly into the uterine horn to avoid in vitro embryo manipulation which may affect the embryo survival.

In humans, functional analogues of rodent specific Crry, CD46 and CD55 play crucial role in the regulation of initial steps of complement cascade. Decreased expression of these proteins on placenta or loss of function mutations in them would increase complement activation causing elevated systemic C3a and increased cellular deposition of MAC. Several recent studies indicating elevated C3a, increased placental MAC deposition and association between CD46 mutations and preeclampsia suggest that in preeclampsia patients complement activation is increased at the feto-maternal interface possibly secondary to defective regulation [15,21,23]. Since new onset of blood pressure increase during third trimester of pregnancy is a hallmark of preeclampsia, increased blood pressure in pregnant mice upon placental complement activation after Crry downregulation suggests that placental complement regulation plays an important role in the pathophysiology of preeclampsia and associated fetal growth restriction.

The magnitude of complement activation on placenta determined by the level of Crry expression may dictate the phenotype. In our mouse model we were able to alter the placental Crry levels using different doxycycline doses (S1 Fig). Placental Crry levels were reduced by about 70% with a high dose (500 μg/mL) of doxycycline resulting in increased embryo resorption. We observed about 30% Crry downregulation and a minimal embryo loss with the doxycycline dose of 75 μg/mL which was used in subsequent experiments. The difference in embryo survival can be attributed to the level of Crry since consistently equal lentiviral titers were used in these experiments. In addition, the doxycycline dose appear to dictate the location of Crry downregulation within placenta. With 75 μg/mL of doxycycline the Crry downregulation was observed only in the labyrinthine zone but not in the junctional zone. The cause of zonal specific effect of doxycycline may not be temporal or related to mode of administration since in mice, it has been shown that plasma concentrations of doxycycline increases rapidly and reaches plateau in 2 days when the doxycycline was given through drinking water [34]. In our study mice were provided with doxycycline containing water starting from 10.5 dpc until 17.5 dpc and therefore, the duration was sufficient for plasma concentration to reach plateau. However, when doxycycline was provided in the drinking water, the final plasma concentration at plateau was directly related to the dose [35]. It is possible that higher plasma concentrations of doxycycline allows it to drain deep into placental tissue and thus, distribute to both the zones. On the other hand low to moderate doses of doxycycline (25 and 75 μg/mL) in drinking water may result in its draining into the proximal labyrinthine zone only. Therefore, we speculate that the zonal specific Crry downregulation was an effect of doxycycline dose. Taken together, this mouse model may allow us to attain a range of complement activation on placenta in a zone specific manner by varying doxycycline dose in drinking water.

The amplitude of placental complement activation may determine the severity of preeclampsia. Preeclampsia is a heterogeneous disease with a range of manifestations from mild to severe. Furthermore, preeclampsia affects fetal growth in a manner related to the severity of the disease. Severe and early onset preeclampsia was found to be associated with a 12–23% reduction in birth weight [36]. Severe preeclampsia is primarily defined by *systolic BP of ≥160 mm Hg or diastolic BP ≥110 mm Hg and proteinuria (≥ 300 mg/24 hours*, *≥ 0*.*3 mg/dL Protein/creatinine or dipstick reading of 2+) or in the absence of proteinuria any one of the following*: *thrombocytopenia (Platelet < 100*,*000 x 10*^*9*^*/L)*, *serum creatinine >1*.*1 mg/dL*, *liver transaminases to twice the normal concentration*, *pulmonary edema* [37]. It is not possible to compare BP measurements between mice and human to ascertain if placental Crry downregulation using 75 μg/mL doxycycline resulted in severe preeclampsia. However, in our study we observed around 20% decrease in fetal weight upon placental Crry downregulation using 75 μg/mL doxycycline suggesting that this dose produced a severe type of preeclampsia. Nevertheless, our mouse model will be useful to ascertain if placental complement activation is directly related to the severity of preeclampsia.

## Conclusions

The data presented in this study suggested that increased complement activation related to the decreased placental complement regulatory protein play a role in the pathogenesis of preeclampsia -like symptoms, more akin to that in humans. Further characterization is underway to investigate if these mice also develop several other features observed in preeclampsia patients such as angiogenic imbalance, inflammatory biomarkers, glomerular endotheliosis and proteinuria, defective spiral artery remodeling, etc. The mechanisms that link complement mediated placental pathology to fetal growth and maternal blood pressure regulation will also need to be addressed in future studies. The data presented in this study clearly showed that placental Crry is critical for normal fetal growth and for maintaining normal blood pressure.

## Supporting information

S1 Fig Embryo survival upon induction of shRNA with different doses of doxycycline (n = 3). (PPTX)

S2 Fig Average litter size at 17.5 dpc in CrryshRNA and control mice. (PPTX)

S1 Raw image (TIF)

S2 Raw image (TIF)

S3 Raw image (TIF)

S4 Raw image (TIF)

S5 Raw image (TIF)

S6 Raw image (TIF)

S7 Raw image (TIF)

S8 Raw image (TIF)

S9 Raw image (TIF)
