# Supplementary material for: Complement inhibitor Crry expression in mouse placenta is essential for maintaining normal blood pressure and fetal growth
Source: PLoS One. 2020 Aug 3;15(8):e0236968. doi: 10.1371/journal.pone.0236968 (PMC7398533; doi:10.1371/journal.pone.0236968)
Supplement: S1 Fig — (PPTX) [file pone.0236968.s001.pptx]

## Slide 1
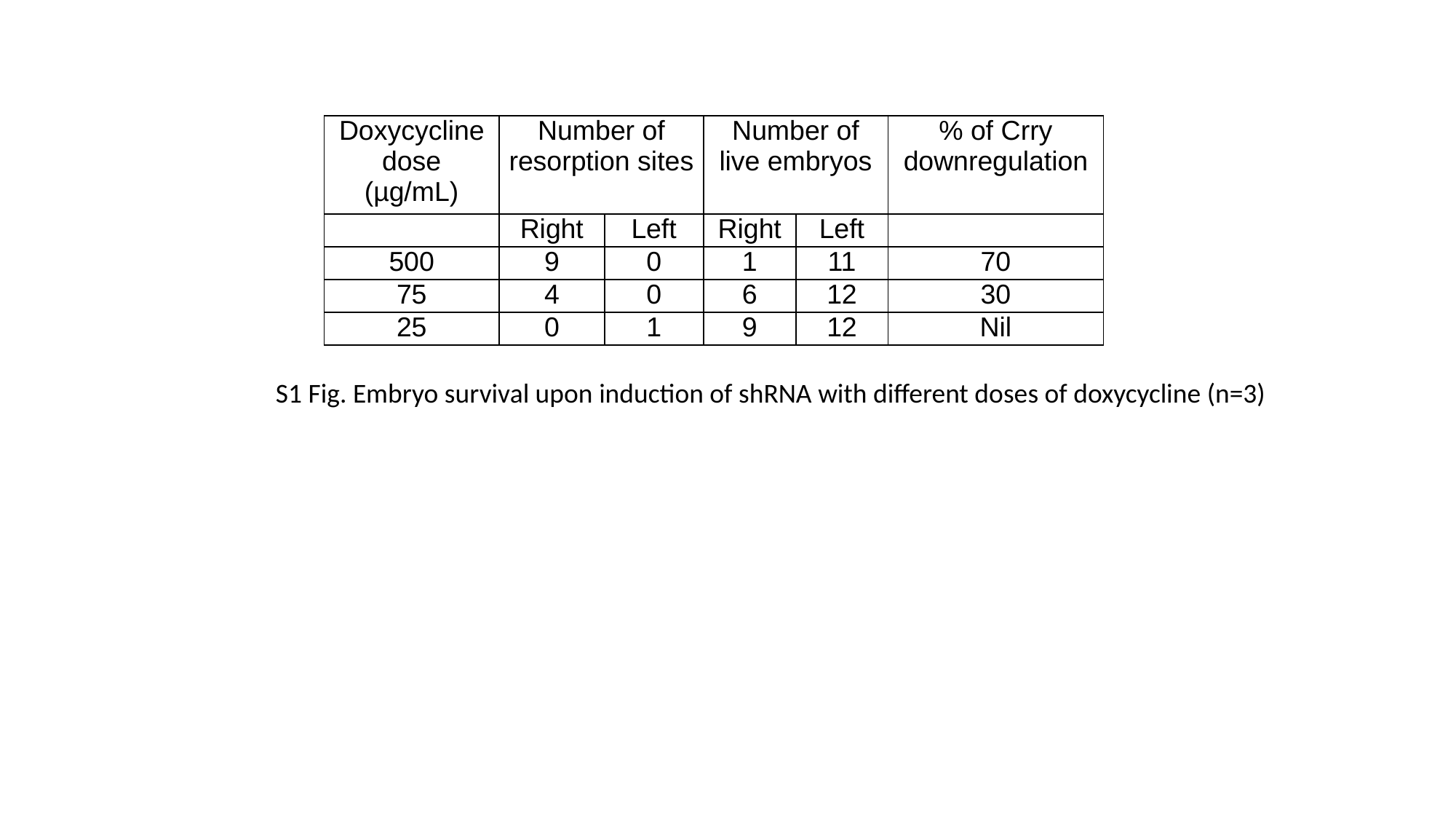

| Doxycycline dose (µg/mL) | Number of resorption sites | | Number of live embryos | | % of Crry downregulation |
| --- | --- | --- | --- | --- | --- |
| | Right | Left | Right | Left | |
| 500 | 9 | 0 | 1 | 11 | 70 |
| 75 | 4 | 0 | 6 | 12 | 30 |
| 25 | 0 | 1 | 9 | 12 | Nil |
S1 Fig. Embryo survival upon induction of shRNA with different doses of doxycycline (n=3)
