# Supplementary figures and images for: Complement inhibitor Crry expression in mouse placenta is essential for maintaining normal blood pressure and fetal growth
Source: PLoS One. 2020 Aug 3;15(8):e0236968. doi: 10.1371/journal.pone.0236968 (PMC7398533; doi:10.1371/journal.pone.0236968)

## Slide 1
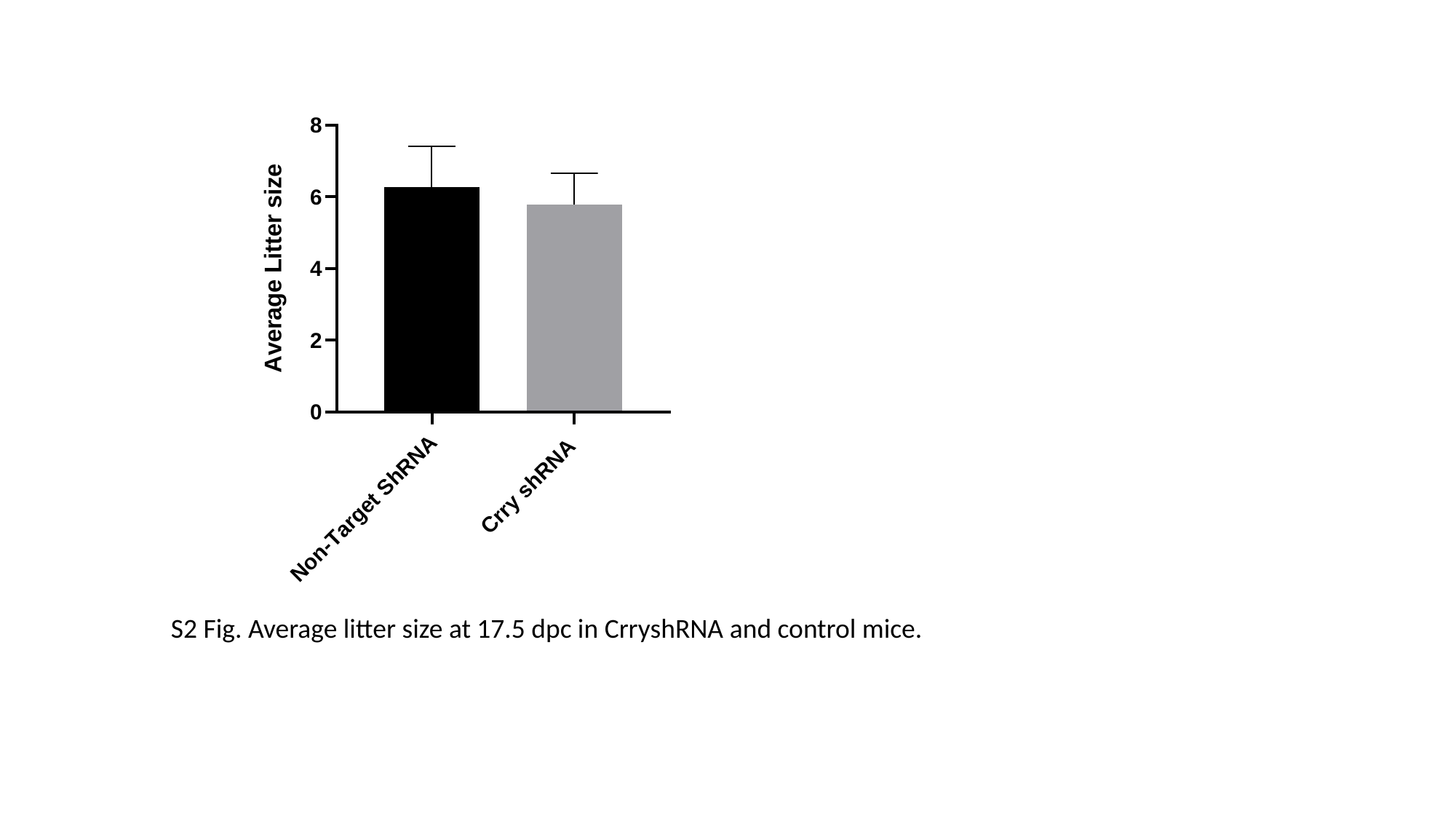

S2 Fig. Average litter size at 17.5 dpc in CrryshRNA and control mice.

Supplement: S2 Fig — (PPTX) [file pone.0236968.s002.pptx]

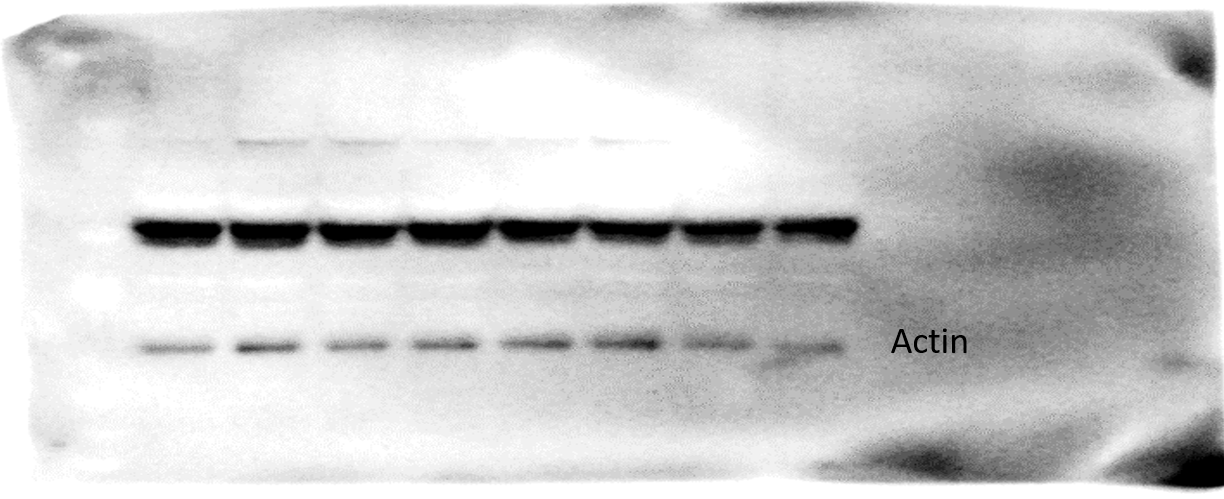

Supplement: S1 Raw image — (TIF) [file pone.0236968.s003.tif]

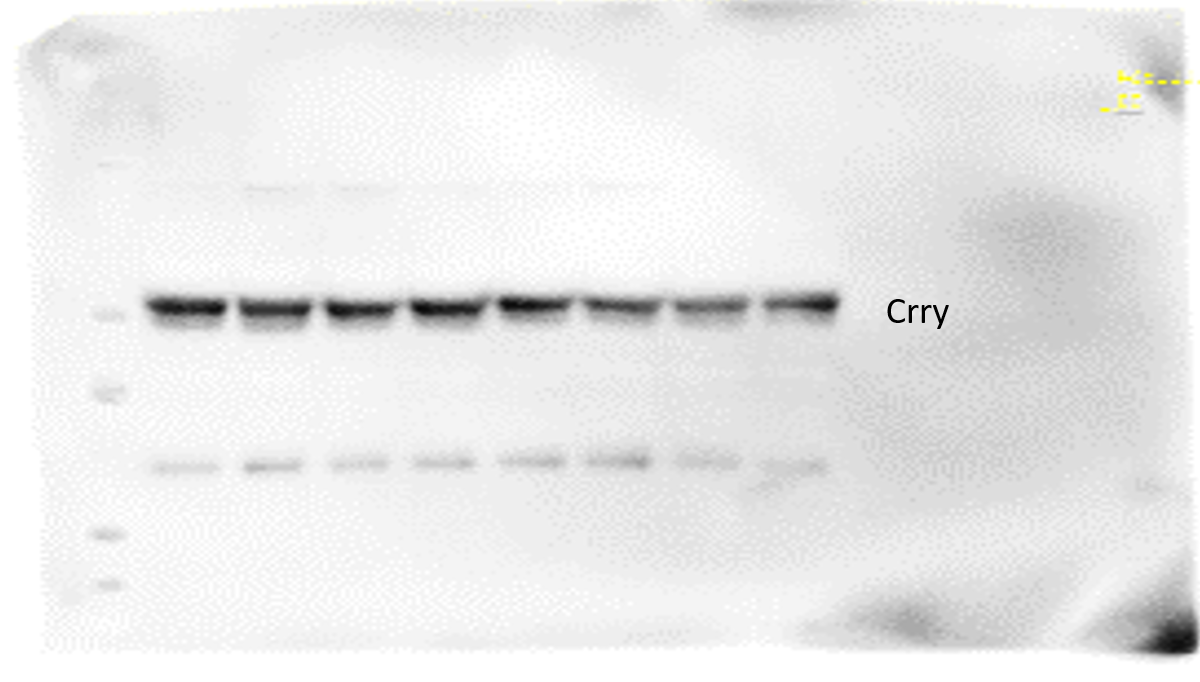

Supplement: S2 Raw image — (TIF) [file pone.0236968.s004.tif]

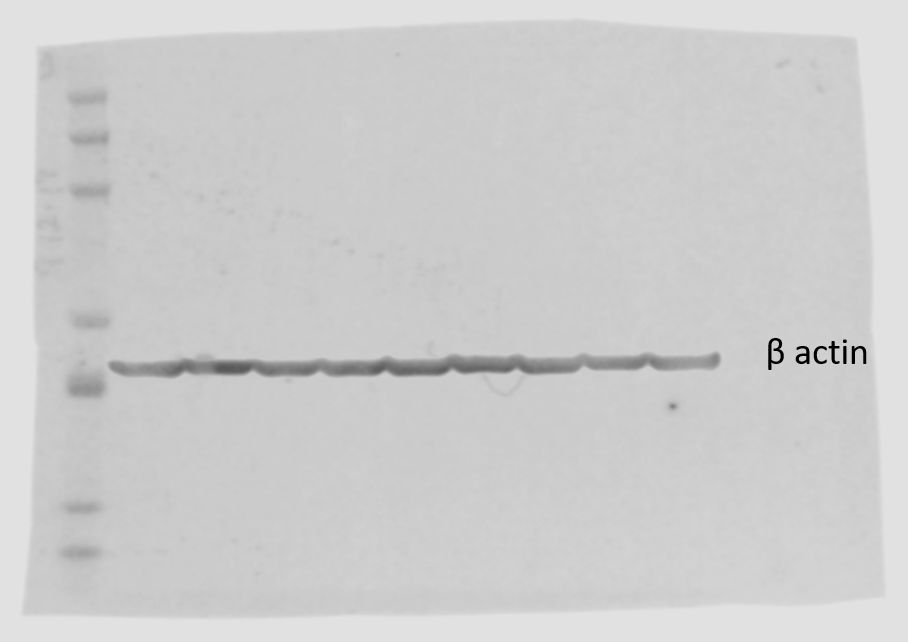

Supplement: S3 Raw image — (TIF) [file pone.0236968.s005.tif]

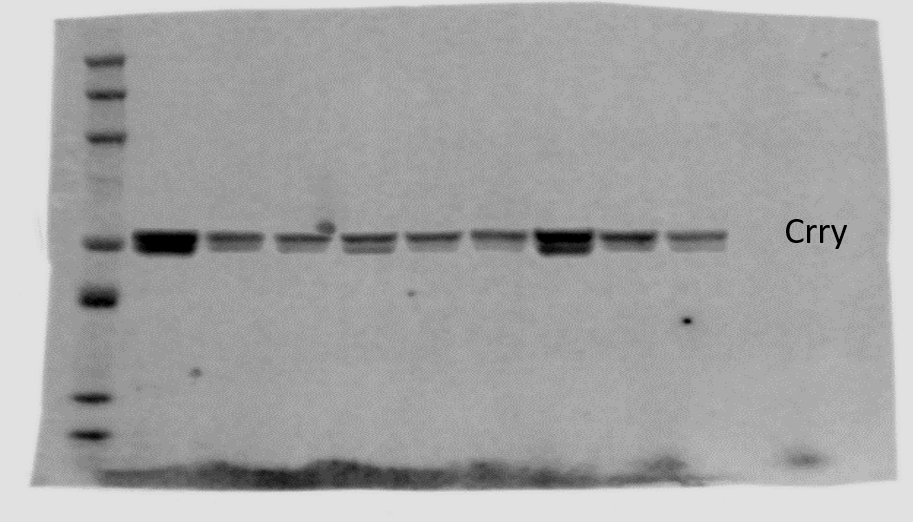

Supplement: S4 Raw image — (TIF) [file pone.0236968.s006.tif]

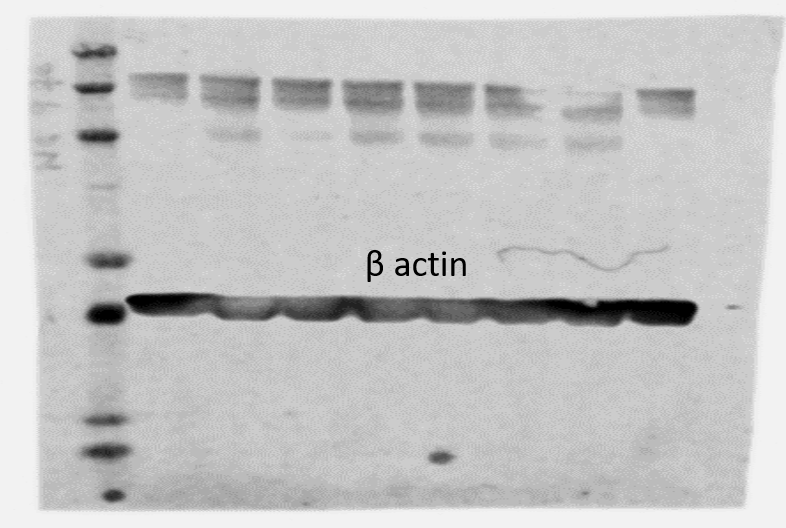

Supplement: S5 Raw image — (TIF) [file pone.0236968.s007.tif]

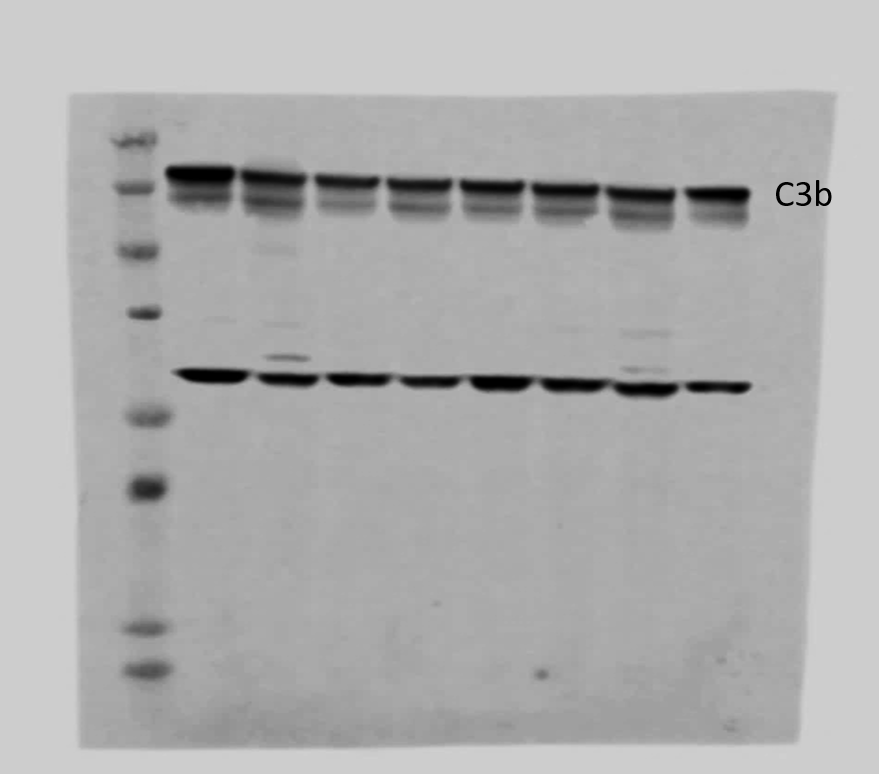

Supplement: S6 Raw image — (TIF) [file pone.0236968.s008.tif]

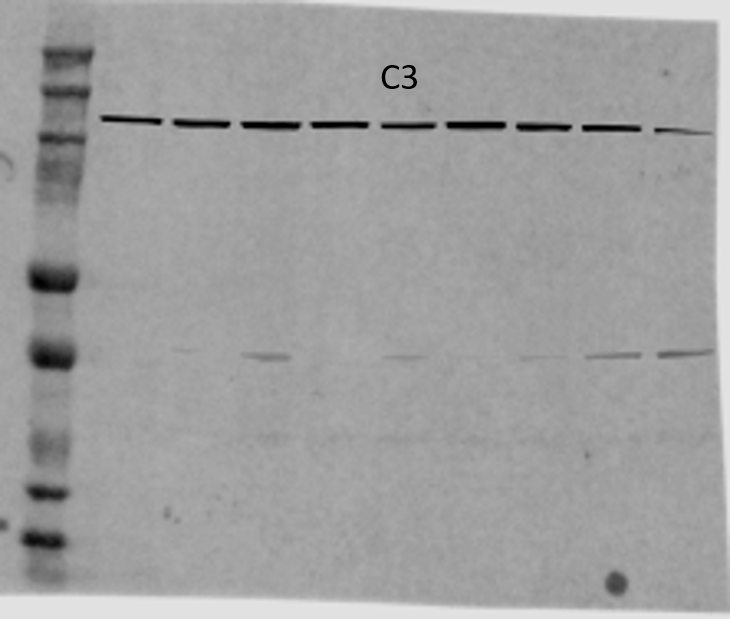

Supplement: S7 Raw image — (TIF) [file pone.0236968.s009.tif]

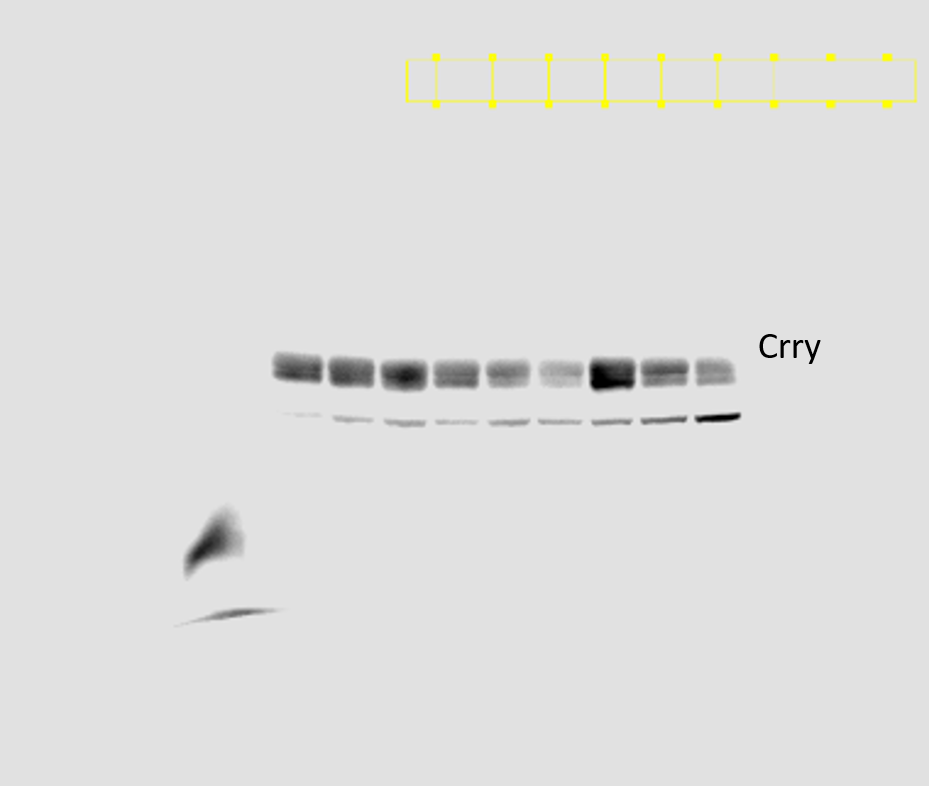

Supplement: S8 Raw image — (TIF) [file pone.0236968.s010.tif]

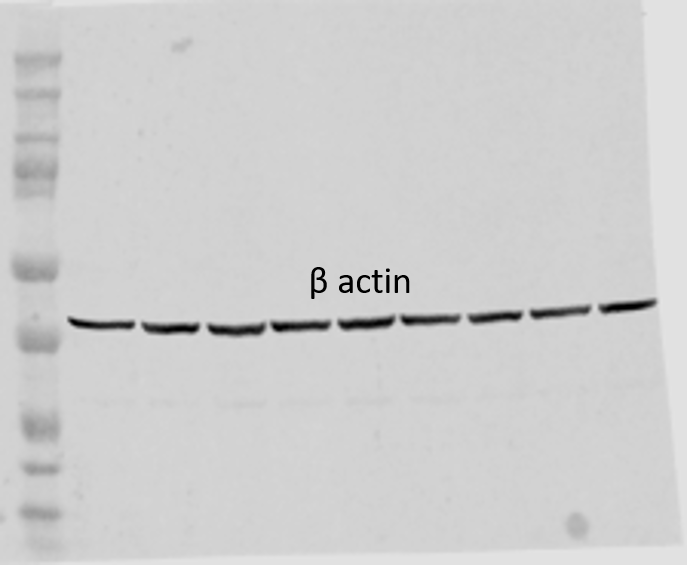

Supplement: S9 Raw image — (TIF) [file pone.0236968.s011.tif]
